# Fathers’ preconception smoking and offspring DNA methylation

**DOI:** 10.1186/s13148-023-01540-7

**Published:** 2023-08-31

**Authors:** Negusse Tadesse Kitaba, Gerd Toril Mørkve Knudsen, Ane Johannessen, Faisal I. Rezwan, Andrei Malinovschi, Anna Oudin, Bryndis Benediktsdottir, David Martino, Francisco Javier Callejas González, Leopoldo Palacios Gómez, Mathias Holm, Nils Oskar Jõgi, Shyamali C. Dharmage, Svein Magne Skulstad, Sarah H. Watkins, Matthew Suderman, Francisco Gómez-Real, Vivi Schlünssen, Cecilie Svanes, John W. Holloway

**Affiliations:** 1 Human Development and Health, Faculty of Medicine, University of Southampton, Southampton, SO16 6YD UK; 2 Department of Clinical Sciences, University of Bergen, Bergen, Norway; 3 Department of Occupational Medicine, Haukeland University Hospital, Bergen, Norway; 4 Centre for International Health, Department of Global Public Health and Primary Care, University of Bergen, Bergen, Norway; 5 Department of Computer Science, Aberystwyth University, Aberystwyth, UK; 6 Department of Medical Sciences: Clinical Physiology, Uppsala University, Uppsala, Sweden; 7 Section of Sustainable Health, Department of Public Health and Clinical Medicine, Umeå University, Umeå, Sweden; 8 Department of Allergy, Respiratory Medicine and Sleep, Landspitali University Hospital, Reykjavik, Iceland; 9 Faculty of Medicine, University of Iceland, Reykjavik, Iceland; 10 Wal-Yan Respiratory Research Centre, Telethon Kids Institute, University of Western Australia, Perth, Australia; 11 Department of Pulmonology, Albacete University Hospital Complex, Albacete, Spain; 12 El Torrejón Health Centre, Andalusian Health Service, Huelva, Spain; 13 Occupational and Environmental Medicine, School of Public Health and Community Medicine, Institute of Medicine, University of Gothenburg, Gothenburg, Sweden; 14 Centre for Epidemiology and Biostatistics, Melbourne School of Population and Global Health, University of Melbourne, Melbourne, Australia; 15 University of Bristol, MRC Integrative Epidemiology Unit, Population Health Sciences, Bristol Medical School, Bristol, UK; 16 Department of Gynaecology and Obstetrics, Haukeland University Hospital, Bergen, Norway; 17 Department of Public Health, Work, Environment and Health, Danish Ramazzini Centre, Aarhus University Denmark, Aarhus, Denmark; 18 National Research Center for the Working Environment, Copenhagen, Denmark; 19 NIHR Southampton Biomedical Research Center, University Hospitals Southampton, Southampton, UK

**Keywords:** Preconception, Paternal effects, Tobacco smoke, Epigenetic, Epigenome-wide association study, DNA methylation, RHINESSA

## Abstract

**Background:**

Experimental studies suggest that exposures may impact respiratory health across generations via epigenetic changes transmitted specifically through male germ cells. Studies in humans are, however, limited. We aim to identify epigenetic marks in offspring associated with father’s preconception smoking.

**Methods:**

We conducted epigenome-wide association studies (EWAS) in the RHINESSA cohort (7–50 years) on father’s any preconception smoking (*n* = 875 offspring) and father’s pubertal onset smoking < 15 years (*n* = 304), using Infinium MethylationEPIC Beadchip arrays, adjusting for offspring age, own smoking and maternal smoking. EWAS of maternal and offspring personal smoking were performed for comparison. Father’s smoking-associated dmCpGs were checked in subpopulations of offspring who reported no personal smoking and no maternal smoking exposure.

**Results:**

Father’s smoking commencing preconception was associated with methylation of blood DNA in offspring at two cytosine-phosphate-guanine sites (CpGs) (false discovery rate (FDR) < 0.05) in *PRR5* and *CENPP*. Father’s pubertal onset smoking was associated with 19 CpGs (FDR < 0.05) mapped to 14 genes (*TLR9*, *DNTT*, *FAM53B*, *NCAPG2*, *PSTPIP2*, *MBIP*, *C2orf39*, *NTRK2*, *DNAJC14*, *CDO1*, *PRAP1*, *TPCN1*, *IRS1* and *CSF1R*). These differentially methylated sites were hypermethylated and associated with promoter regions capable of gene silencing. Some of these sites were associated with offspring outcomes in this cohort including ever-asthma (NTRK2), ever-wheezing (DNAJC14, TPCN1), weight (FAM53B, NTRK2) and BMI (FAM53B, NTRK2) (*p* < 0.05). Pathway analysis showed enrichment for gene ontology pathways including regulation of gene expression, inflammation and innate immune responses. Father’s smoking-associated sites did not overlap with dmCpGs identified in EWAS of personal and maternal smoking (FDR < 0.05), and all sites remained significant (*p* < 0.05) in analyses of offspring with no personal smoking and no maternal smoking exposure.

**Conclusion:**

Father’s preconception smoking, particularly in puberty, is associated with offspring DNA methylation, providing evidence that epigenetic mechanisms may underlie epidemiological observations that pubertal paternal smoking increases risk of offspring asthma, low lung function and obesity.

**Supplementary Information:**

The online version contains supplementary material available at 10.1186/s13148-023-01540-7.

## Introduction

There is growing consensus that perturbations of the epigenome through parental exposures even before their offspring are conceived may explain some of the variation in the heritability of health and disease not captured by genome-wide association studies (GWAS). The period of puberty in future parents, in particular fathers, may represent a critical window of physiological change and epigenetic reprogramming events, which may increase the individual’s susceptibility for environmental exposures to be embodied in the developing gametes [1, 2]. Animal and human studies have shown that prenatal as well as personal exposure to smoking are associated with epigenetic modifications that impact on sperm count and quality [3]. There is now growing interest in how epigenetic modifications, such as DNA methylation (DNAm), related to the parental *preconception* period may influence the health of the *next generation* [4].

Although smoking rates are generally declining, smoking commencing before the age of 15 is increasing in European countries [5, 6]. Epidemiological studies have demonstrated that father’s smoking in adolescent years may be a causal factor for poorer respiratory health in offspring. Both fathers’ smoking initiation before age 15 and smoking duration before conception have been associated with more asthma and lower lung function in offspring [7–9]. Father’s preconception smoking onset has also been associated with higher body fat mass in sons [10–13].

Epigenome-wide association studies (EWAS) have identified extensive methylation biomarkers associated with personal smoking [14], all-cause mortality in current and former smokers, as well as mother’s smoking during pregnancy [15–17]. While previous studies have identified DNA methylation signals in offspring blood [16] and cord blood [17] related to father’s smoking, they have not specifically investigated the timing of exposure, partly because detailed smoking information from fathers is rarely available [18]. Methylation markers associated with paternal preconception smoking, could have an important role in elucidating long-term effects on the offspring epigenome, with the potential for developing efficient intervention programmes and improved public health.

This study aimed to investigate whether DNA methylation of DNA measured in offspring blood is associated with fathers’ smoking commencing before conception, and in particular, with fathers’ smoking starting in (pre)pubertal years (before age 15). We hypothesized that epigenetic changes involving DNA methylation may explain the molecular mechanisms underlying the association between fathers’ smoking preconception and offspring health observed in epidemiological studies. Additionally, we hypothesized that fathers’ smoking in the critical window of early puberty, as compared to smoking initiated at a later age, may have a more significant impact on the offspring epigenome. In a two-generation cohort, we sought to identify the DNA methylation changes in offspring blood (aged 7–50 years) associated with (1) father’s smoking onset preconception compared with never or later onset smoking and (2) father’s smoking onset before age 15 compared with never smoking. Finally, given the range of epidemiological studies reporting sex-specific outcomes in the offspring [10, 19], we wanted to explore whether patterns of associations between fathers’ preconception smoking and offspring DNA methylation were different for sons and daughters.

## Methods

### Study design and data

We used data and samples from offspring, aged 7–50 years that participated in the RHINESSA study (www.rhinessa.net). Parent data, including detailed information on smoking habits, were retrieved from the population-based European Community Respiratory Health Survey (ECRHS, www.ecrhs.org) and/or the Respiratory Health in Northern Europe study (RHINE, www.rhine.nu) studies. This analysis comprised 875 offspring-parent pairs with complete information, from six study centres with available peripheral blood for offspring (Aarhus, Denmark; Albacete/Huelva, Spain; Bergen, Norway; Melbourne, Australia; Tartu, Estonia). All participants were of Caucasian ancestry. Medical research committees in each study centre approved the studies, and each participant gave written consent. The ethical approval reference numbers are listed on www.rhinessa.net.

Father’s smoking and age of starting/quitting were reported in interviews/questionnaires and related to offspring’s birth year, to define the categories: never smoked (*n* = 547), any preconception smoking (*n* = 328), preconception smoking with onset < 15 years (pubertal smoking) (*n* = 64) (cut point based on mean age of voice break 14.5 years, first nocturnal seminal emission 14.8 years). Personal smoking was classified as current, ex- or never smoking. Maternal smoking was defined by offspring’s report of mothers’ smoking during their childhood/pregnancy.

#### Methylation profiling and processing

DNAm in offspring was measured in 1µg of DNA extracted from peripheral blood, using a simple salting out procedure [20]. Bisulphite conversion was undertaken using EZ 96-DNA methylation kits (Zymo Research, Irvine, CA, USA) at the Oxford Genomics Centre (Oxford, UK) and methylation assessed using Illumina Infinium MethylationEPIC Beadchip arrays (Illumina, Inc. CA, USA) with samples randomly distributed on microarrays to control against batch effects.

Data analysis was undertaken using Statistical Computing Program R, version 3.6.1 [21]. Methylation intensity files were processed and quality was assessed using minfi [22] and Mefil [23]**.** Methylation distribution for outliers was assessed using density and multidimensional scaling plots, methylated vs unmethylated ratio plot, sex mismatch and sex outliers, control probes and bisulphite conversion efficiency. Normalization was carried out using BMIQ [24], which adjusts the intra-sample beta-values of type 2 design and type 1 probes**.** To remove technical variation detected at *p* value 1 × 10^–10^ by the champ.SVD function within the CHAMP package [25], ComBat from SVA [25] was applied on sample batch and slide.

Probes were excluded from analysis using the following criteria: probes with a detection *p* value above 0.01 in one or more samples (*n* = 27,206 probes), probes with a beadcount < 3 in at least 5% of samples (*n* = 1451), non-cg probes (*n* = 2580), probes with SNPs as identified in Zhou et al*.* [26] (*n* = 92,403), probes with multiple hybridization locations as identified in Nordlund et al*.* [27] (*n* = 51) and probes on the X or Y (*n* = 15,776) chromosome and cross-reactive probes on epic array (*n* = 2368) as identified by Pidsley et al [28]. A total of 724,292 probes were used for downstream analysis. Cell-type proportions were estimated using EpiDISH (Epigenetics Dissection of Intra-Sample Heterogeneity) [29].

### Statistical analysis

We ran two EWAS on preconception father’s smoking as exposure (any preconception smoking and prepuberty smoking) with DNA methylation as outcome. To identify differentially methylated cytosine-phosphate-guanine (CpG) sites (dmCPG), robust multiple linear regression models were applied on beta-values using limma [30] adjusting for offspring’s sex, age, personal and mother’s smoking and cell-type proportions (B cells, natural killer cells, CD4 T cells, CD8 T cells, monocyte, neutrophils) at significance level of false discovery rate (FDR) [31] corrected *p* value  < 0.05. Eosinophils were not included due to a very low estimate and to avoid potential multicollinearity. In additional analyses, associations between fathers’ any preconception smoking and offspring’s DNA methylation were also stratified by offspring sex.

Manhattan plots were generated using qqman [32] and a circos plot with CMplot R package [33]. Inflation from systematic biases was adjusted using BACON [34]. Differentially methylated regions were detected using dmrff [35] and DMRCate [36]. Transcription factor binding site prediction was performed using eFORGE TF [37]. Gene-disease association was identified using open target [38]. Identified dmCpGs were compared against EWAS atlas for association with known biological traits [39]. To gain biological insight regarding the dmCpGs mapped to genes, gene interactors were identified using String [40] and enrichment was performed using UniprotR [41] and gometh [42]. Biological interpretation of significant differentially methylated CpGs (dmCpGs) is detailed in the supplementary methods.

To further investigate whether the identified dmCpGs were associated with respiratory outcomes and weight in the offspring, we conducted regression analysis between offspring’s DNA methylation signals and offspring’s own reports of ever-asthma, ever-wheeze, weight and BMI, while accounting for offspring sex.

We constructed two additional EWAS on offspring personal as well as maternal smoking to assess the shared count and overlap of dmCpGs (FDR < 0.05) between each EWAS and to allow for comparison with dmCpGs identified as related to father’s preconception and pubertal smoking. To address potential confounding by offspring personal smoking and maternal smoking, association of the detected dmCpGs was also checked in subpopulations of offspring who reported no personal smoking exposure and offspring with no maternal smoke exposure.

Our EWAS results were also compared with findings from meta-analyses of EPIC array DNA methylation associated with personal smoking from four population-based cohorts [43], personal smoking-methylation effects from 16 cohorts using 450K arrays [14] and the Pregnancy and Childhood Epigenetics Consortium (PACE) meta-analysis of mother’s smoking in pregnancy on offspring cord blood methylation [15].

#### Replication analysis

Replication was carried out in the ALSPAC (Avon Longitudinal Study of Parents and Children) cohort adjusted for predicted cell count proportions, batch effects (plate), maternal smoking during pregnancy, self-reported own smoking, age and sex using DNA methylation data from whole blood measured at age 15–17. A description of the ALSPAC cohort is provided in the supplementary methods. T tests were used to compare the association of regression coefficient of RHINESSA’s dmCpG sites at FDR < 0.05 and the top 100 CpG sites with ALSPAC. Signed tests were used to test the direction of association.

#### Sensitivity analyses

To assess whether fathers’ smoking-related dmCpGs were potentially confounded by the effect of social class, father’s educational level, a surrogate measure of socioeconomic background, was used as an independent variable and regressed with the identified to dmCpGs. The impact of offspring’s age was also more extensively investigated in subsequent analyses, by correlating known age-related CpG markers from the RHINESSA EWAS study, with both top CpGs identified as related to fathers’ smoking, as well as to the age of the offspring.

## Results

The analysis included 875 RHINESSA participants (Table 1A), 457 males and 418 females, aged 7 to 50 years. Of these 328 had a father who had ever smoked before conception (father starting smoking before the birth year of offspring minus 2 years) of which 64 had started before age 15 years; 263 had a mother who had ever smoked, and 240 had smoked themselves. Characteristics are also given for the sub-sample of 304 offspring whose father either had started smoking before age 15 years or never smoked (before or after conception of the offspring) (Table 1B).

**Table 1:** **A** and **B** General characteristics of study participants from the RHINESSA study with complete data on offspring DNA methylation and father’s age of onset of tobacco smoking

Characteristic	A: Any preconception smoking onset			B: Fathers smoking onset < 15 years		
	No*, *N* = 547	Yes, * N* = 328	*p* value^1^	No, * N* = 240	Yes, * N* = 64	*p* value^2^
*Sex*, n (%) Female Male	263 (48) 284 (52)	155 (47) 173 (53)	0.8	112 (47) 128 (53)	33 (52) 31 (48)	0.5
*Study centre*, n (%) Albacete Arhus Bergen Huelva Melbourne Tartu	24 (4) 34 (6) 320 (59) 17 (3) 78 (14) 74 (14)	39 (10) 17 (5) 194 (59) 14 (4) 14 (4) 57 (17)	< 0.001	7 (3) 14 (6) 174 (72) 5 (2) 21 (9) 19 (8)	9 (14) 1 (2) 47 (73) 2 (3) 1 (2) 4 (6)	0.005
*Age*, mean (SD)	26 (8)	30 (8)	< 0.001	26 (8)	27 (8)	0.2
*Mother smoking*, n (%)	84 (15)	179 (55)	< 0.001	30 (12)	34 (53)	< 0.001
*Offspring smoking*, n (%)	121 (22)	119 (36)	< 0.001	44 (18)	25 (39)	< 0.001
*Cell proportions*, mean (SD)						
B cells	0.02 (0.02)	0.02 (0.02)	0.4	0.02 (0.02)	0.02 (0.02)	0.4
CD4 cells	0.03 (0.03)	0.03 (0.03)	0.3	0.03 (0.03)	0.03 (0.02)	> 0.9
CD8 cells	0.13 (0.05)	0.12 (0.05)	0.5	0.13 (0.04)	0.12 (0.04)	0.7
NK cells	0.07 (0.04)	0.07 (0.04)	0.8	0.07 (0.04)	0.07 (0.04)	0.4
Mononuclear cells	0.07 (0.02)	0.07 (0.02)	0.2	0.07 (0.02)	0.07 (0.02)	0.4
Neutrophil	0.67 (0.09)	0.68 (0.08)	0.3	0.68 (0.08)	0.68 (0.07)	0.8

**A** for the full cohort of 875 offspring whose father started to smoke at any time preconception, and **B** for the 304 offspring whose father started to smoke before age 15 years or never smoked

*No smoking reference category includes all without preconception smoking (father starting smoking before the birth year minus 2 years), i.e. those with fathers who either never smoked or started to smoke after the offspring’s conception. ^1^Pearson’s Chi-squared test; Wilcoxon rank sum test; ^2^Pearson’s Chi-squared test; Fisher’s exact test; Wilcoxon rank sum test

### Epigenome-wide association analysis of preconception father’s smoking

**Fig. 1:**
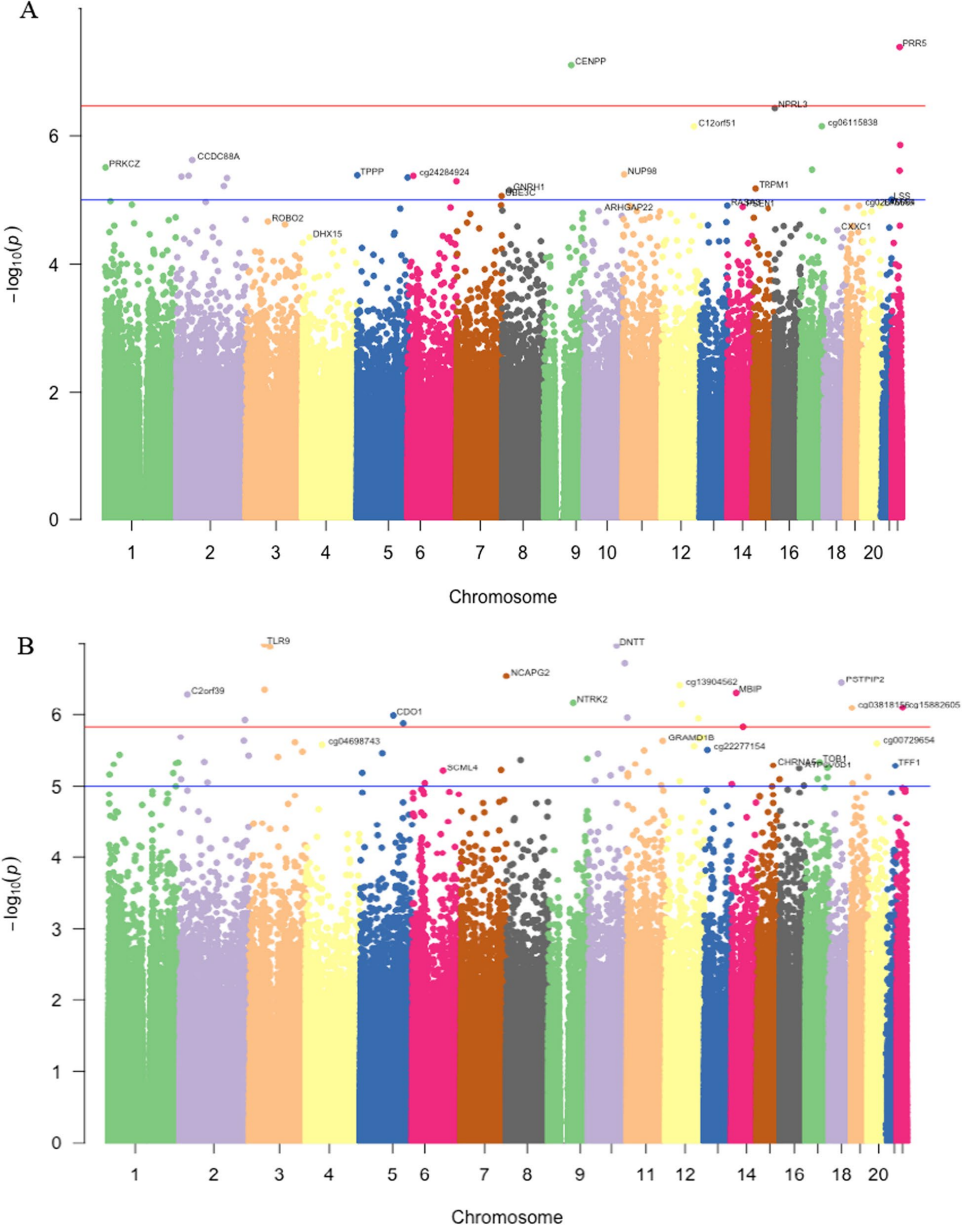
Manhattan plot for genome-wide distribution of dmCpGs. **A**: for father’s any preconception smoking and **B**: father’s pubertal smoking starting before age 15. The red line shows genome-wide significance, the blue is the suggestive line. The y-axis represents − log10 of the *p* value for each dmCpG (indicated by dots) showing the strength of association. The x-axis shows the position across autosomal chromosomes. The top dmCpGs on each chromosome were annotated to the closest gene

**Fig. 2:**
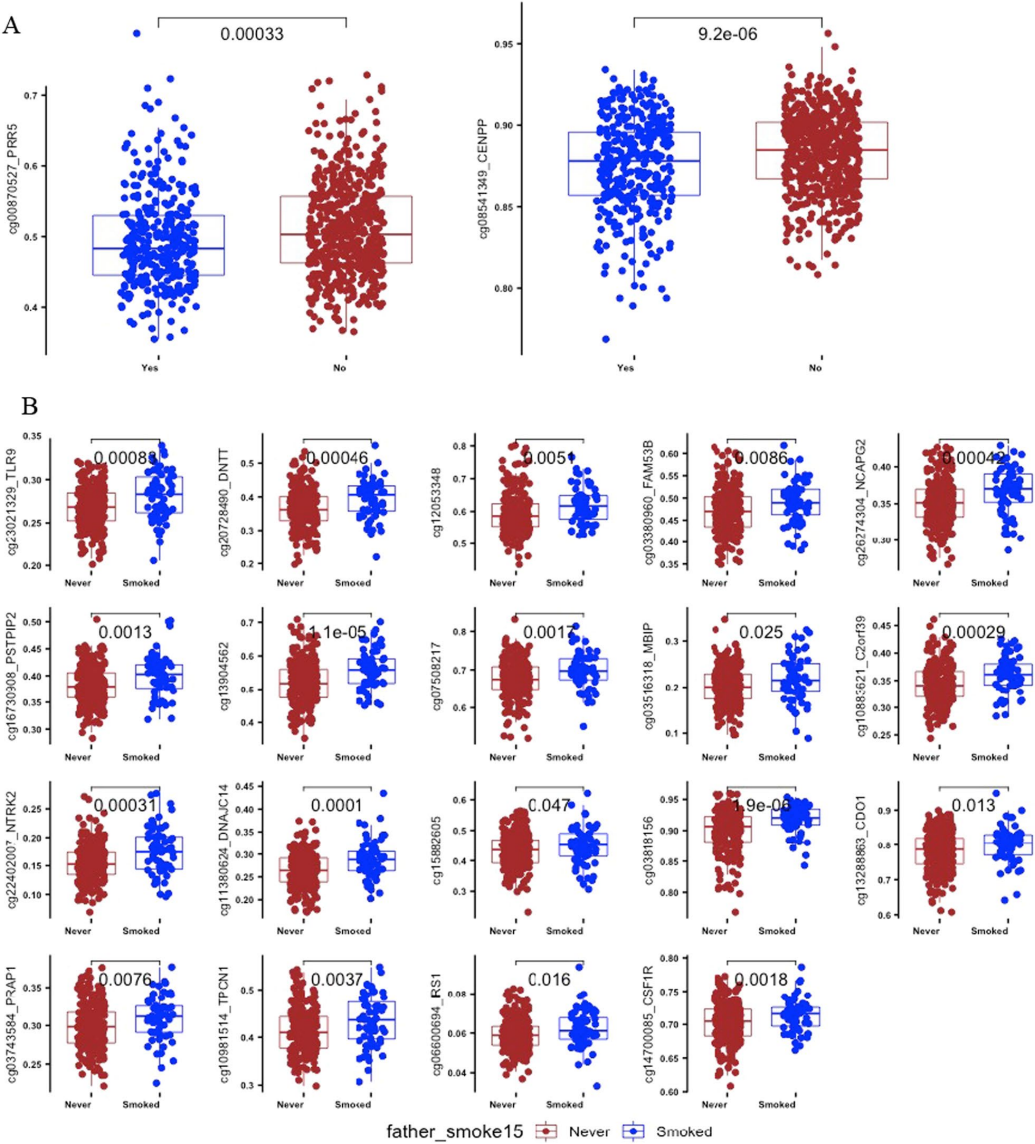
Box plots showing distribution of methylation levels (beta-values) by significant dmCpGs sites in the EWAS. **A**: father’s any preconception smoking and **B**: for father’s pubertal smoking starting before age 15. The comparison *p* value between never-smoking exposed and smoking exposed offspring is shown above the box plot for each dmCpG

Epigenome-wide association between father’s any preconception smoking and offspring DNA methylation identified two dmCpGs (inflation λ = 1.187); cg00870527 mapped to *PRR5* and cg08541349 mapped to *CENPP* (Table 2A and Additional file 1: table E1). The genome-wide distribution of associated dmCpGs is shown in Fig. 1A. Figure 2A shows a comparison of methylation beta-values between the never- and ever-smoked groups for two CpG sites. In both cases, the methylation values are significantly lower in the offspring of ‘ever-smoked’ fathers; cg00870527 in *PRR5* (*p* value  = 0.0003) and cg08541349 in *CENPP* (*p* value  = 0.0000092).

**Table 2:** **A** and **B**. CpG sites associated with father’s smoking at genome-wide significance (FDR < 0.05)

Fathers’ smoking	CpG	Coefficient*	Average**	SD	Adj.P***	Gene	Location****
A: Any preconception smoking onset	cg00870527	− 0.024	0.5	0.07	0.028	PRR5	N_Shelf
	cg08541349	− 0.012	0.88	0.023	0.028	CENPP	OpenSea
B: Fathers’ smoking onset before age 15	cg23021329	0.015	0.27	0.021	0.026	TLR9	S_Shore
	cg20728490	0.032	0.37	0.049	0.026	DNTT	OpenSea
	cg12053348	0.036	0.61	0.056	0.026	NA	OpenSea
	cg03380960	0.019	0.48	0.045	0.034	FAM53B	OpenSea
	cg26274304	0.018	0.36	0.027	0.037	NCAPG2	N_Shore
	cg16730908	0.021	0.39	0.032	0.037	PSTPIP2	S_Shore
	cg13904562	0.041	0.53	0.056	0.037	NA	OpenSea
	cg07508217	0.026	0.69	0.042	0.037	NA	OpenSea
	cg03516318	0.028	0.21	0.039	0.037	MBIP	OpenSea
	cg10883621	0.02	0.35	0.032	0.037	C2orf39	Island
	cg22402007	0.022	0.16	0.031	0.041	NTRK2	N_Shore
	cg11380624	0.024	0.27	0.036	0.041	DNAJC14	N_Shore
	cg15882605	0.025	0.44	0.051	0.041	NA	OpenSea
	cg03818156	0.017	0.9	0.028	0.041	NA	OpenSea
	cg13288863	0.02	0.79	0.049	0.048	CDO1	N_Shore
	cg03743584	0.018	0.3	0.025	0.048	PRAP1	OpenSea
	cg10981514	0.023	0.42	0.042	0.048	TPCN1	OpenSea
	cg06600694	0.005	0.06	0.008	0.048	IRS1	Island
	cg14700085	0.016	0.71	0.024	0.050	CSF1R	OpenSea

**A** for father’s any preconception smoking, in the full cohort (*N* = 875), and **B** for father’s smoking starting before age 15 years, in the subpopulation (*N* = 304)

*Coefficient: Regression coefficient between father smoking/not smoking

**Average methylation across all samples

***adj. P. Val: FDR adjusted p value

****N (north) Shelf: up to 2 kb outward from flanking shores; OpenSea: > 4 kb from CpG islands; N (north) and S (south) Shores: up to 2 kb from flanking CpG islands

In sex-stratified analysis, in males (*n* = 457) we identified four dmCpGs mapped to *KCNJ1*, *GRAMD4*, *TRIM2* and *MYADML2*. In females (*n* = 418) there was one dmCpG mapped to LEPROT1 (FDR <  = 0.05) (Additional file 1: Table E2). All sex-specific dmCpGs were hypomethylated.

To specifically determine the signature related to father’s early onset smoking, we compared methylation differences between offspring of fathers who started to smoke < 15 years (*n* = 64) with offspring of never smoking fathers (*n* = 240). We identified 55 dmCpGs at FDR < 0.05 (*λ* = 1.44) showing genome-wide significance. After adjusting for inflation using BACON, 19 dmCpGs showed significant association at FDR < 0.05 with λ = 1.29 (Table 2B, Fig. 1B and Additional file 1: Table E3). These dmCpGs were mapped to 14 known genes and 5 intergenic regions. The genes include *TLR9*, *DNTT*, *FAM35B*, *NCAPG2*, *MBIP*, *C2orf39*, *NTRK2*, *DNAJC14*, *CDO1*, *PRAP1*, *TPCN1*, *IRS1*, *PSTPIP2* and *CF1R*. All hits were hypermethylated in the exposed group. The comparison of methylation distribution between the never and smoke exposed is shown in Fig. 2B.

The dmCpGs associated with father’s preconception smoking were mainly located in open-sea genomic features and enriched for promoter regions (Table 2A). The dmCpGs associated with father’s pubertal smoking were in open-sea genomic features and CpG island shores (flanking shore regions, < 2 kb up-and downstream of CpG islands) and enriched for CpG islands and gene bodies (Table 2B).

### Associations between fathers’ smoking-related dmCpGs and offspring phenotypes

Some of the identified dmCpG sites showed association with ever-asthma (cg22402007: NTRK2), ever-wheezing (cg11380624: DNAJC14, cg10981514: TPCN1), weight (cg12053348, cg03380960: FAM53B, cg22402007: NTRK2 [44]) and BMI (cg03380960: FAM53B, cg12053348, cg22402007: NTRK2) at p < 0.05 as shown in (Additional file 1: Table E4).

### Father’s preconception smoking signatures as compared with signatures of personal and mother’s smoking

We identified 33 dmCpGs related to personal smoking and 14 dmCpGs associated with mother’s smoking (FDR < 0.05) (Additional file 1: Tables E5 and E6, respectively).

**Fig. 3:**
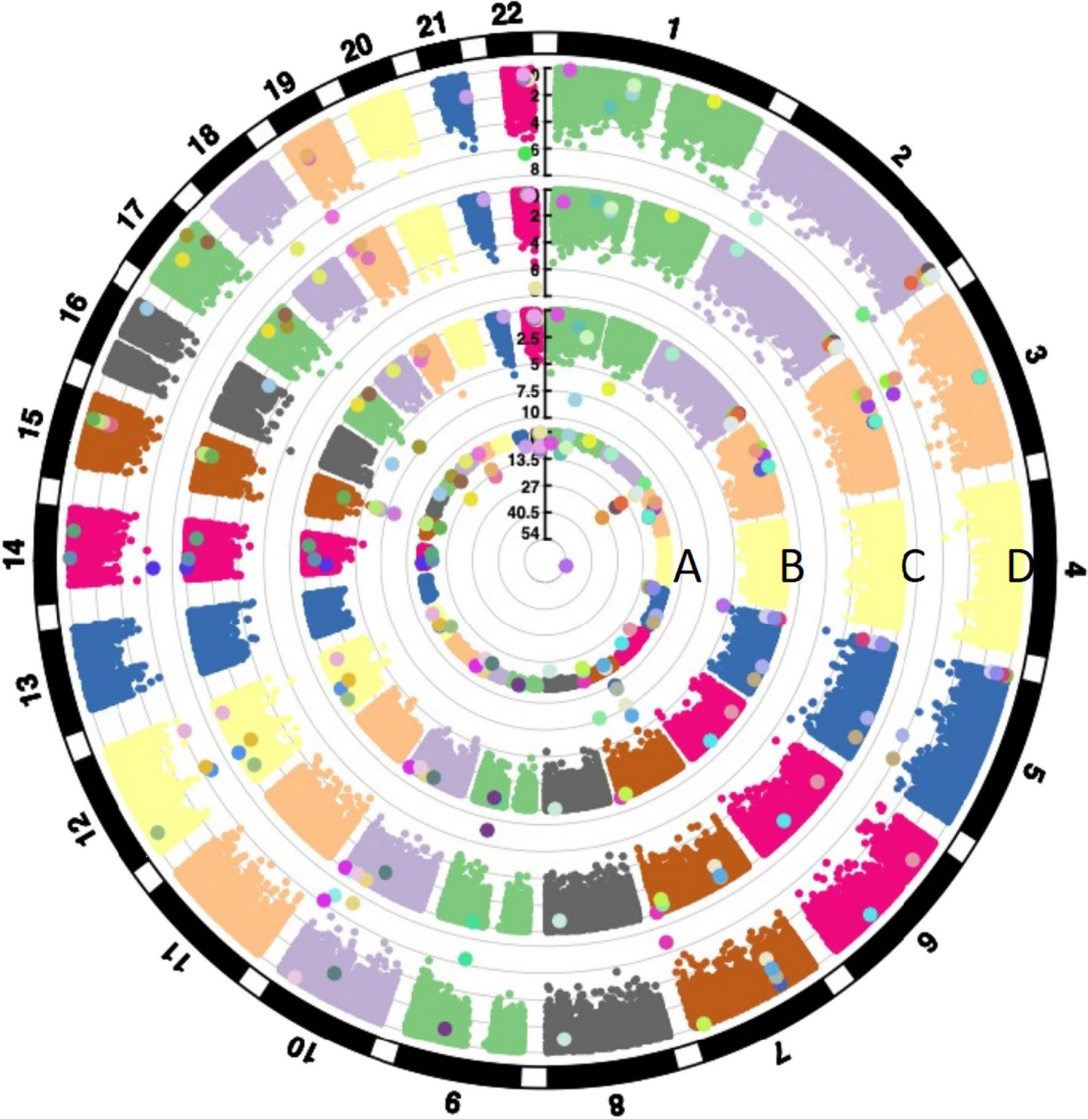
Circos plots showing genome-wide distribution across autosomal chromosomes of dmCpGs associated with **A** personal smoking (in offspring), **B** mother’s smoking, **C** father’s any preconception smoking and **D** father’s pubertal smoking starting before age 15. Each dot represents a CpG site; the radial line shows the − log10 *p* value for each EWAS. Zoomed dots show CpG sites significant in at least one of the EWAS; each zoomed dot colour shows a unique CpG site specific locus

To illustrate the distinct and shared genome-wide effects of personal, mother’s and father’s smoking on the offspring methylome, we generated a locus-by-locus genome comparison (Fig. 3). While there was similarity between the effects of personal smoking and mother’s smoking on chromosome 5, we observed distinct signatures for father’s preconception smoking on chromosome 22 and for mother’s smoking exposure on chromosomes 7 and 15.

To confirm that the father’s smoking-associated dmCpGs were not confounded by offspring’s own or mother’s smoking, we carried out sensitivity analysis in subpopulation of offspring who reported no personal smoking exposure and no maternal smoking exposure. Accounting for all covariates (offspring age, sex, study centre and blood cell type proportions), all paternal smoking-associated dmCpGs remained significant at *p* value  < 0.05 in these analyses (Additional file 1: Table E7). Despite the small sample size (*n* = 175), when we accounted for mothers’ sustained smoking throughout pregnancy as covariate, 13 of 19 identified dmCpGs remained significantly associated with paternal smoking at p value < 0.05 (Additional file 1: Table E7).

**Fig. 4:**
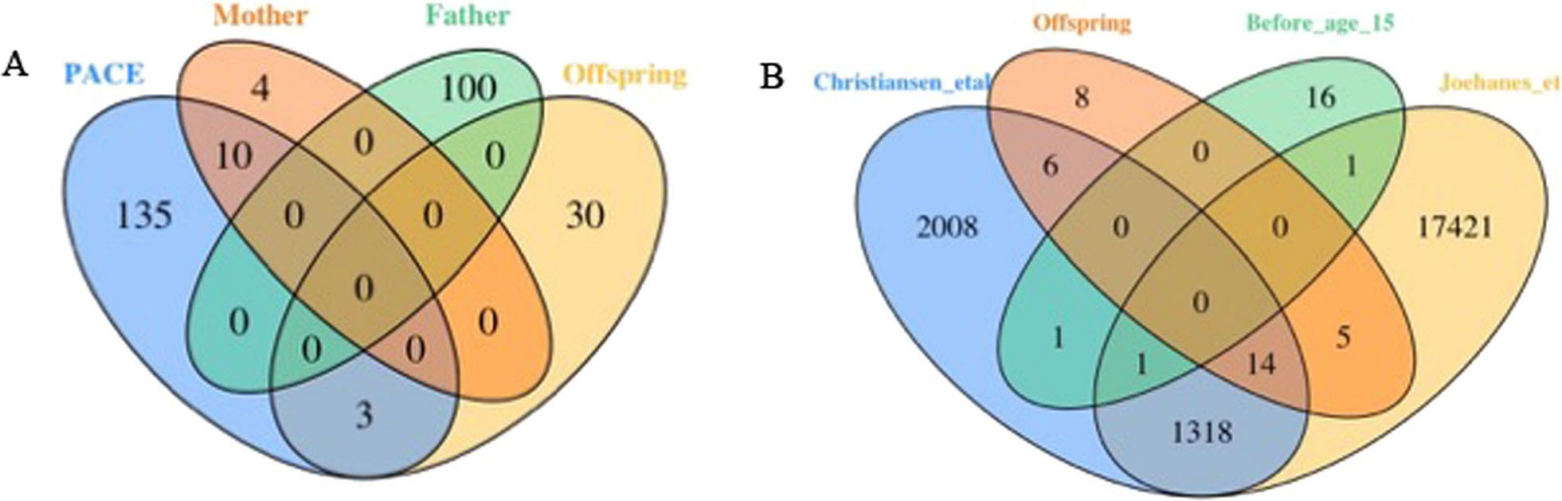
Venn diagram showing EWAS CpG top hits for personal (offspring) smoking, mother’s smoking (FDR < 0.005), father’s any preconception smoking (top 100 dmCpGs) and father’s pubertal smoking starting before age 15 (FDR < 0.05) in the RHINESSA cohort, which are shared with top hits from meta-analysis of **A** PACE mother smoking (blue oval) as reported by Joubert et al. 2016 and **B** Personal cigarette smoking signature as reported by Christiansen et al. 2021 (blue) and by Joehanes et al. 2016 (yellow)

Comparing our EWAS results with previously published meta-analysis results of maternal and personal smoking showed that 16 of our 19 dmCpGs associated with fathers pubertal smoking onset had not previously been associated with maternal or personal smoking [14, 15, 43] (Fig. 4A, B). Nine of the identified CpG sites were also present on the 450k array (Additional file 1: Table E8). Two CpG sites (cg11380624 (*DNAJC14*), cg20728490 (*DNTT*)) were shared with analyses of personal smoking by Joehanes et al. [43] and two sites (cg12053348 (intergenic), cg20728490 (*DNTT*)) with Christiansen et al. [14]. In contrast, 10 of our 14 mother smoking-associated dmCpGs, with 11 CpGs also present at the 450K array, and 25 of our personal smoking-related dmCpGs, with 23 CpGs present at the 450K array, were also reported in the meta-analyses results [14, 15, 43] (Additional file 1: Table E8).

### Enrichment of dmCpGs for related traits

**Fig. 5:**
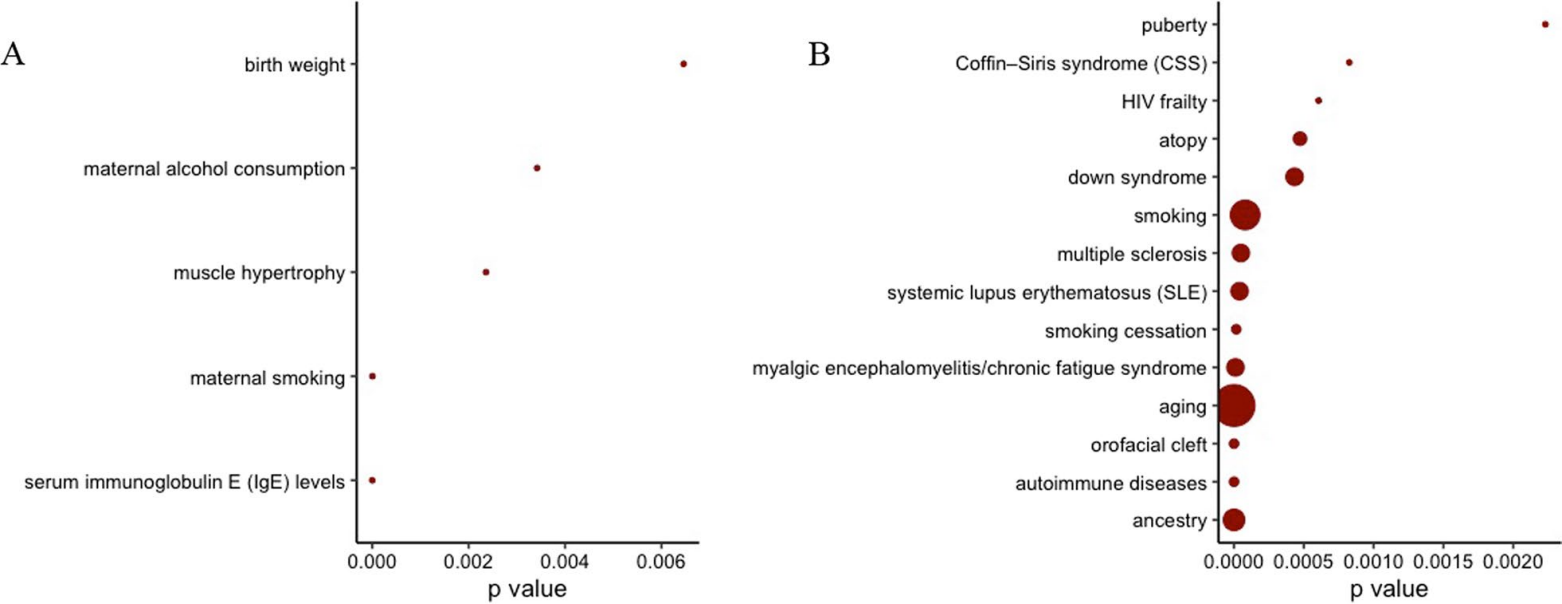
Traits associated with the CpG sites that in EWAS were identified to be differentially methylated according to **A** father’s any preconception smoking, **B** father’s pubertal smoking starting before age 15

We investigated whether the significant dmCpGs associated with father’s preconception smoking onset overlapped with other traits, using the repository of published EWAS literature in the EWAS atlas. The top 23 dmCpG sites for father’s any preconception smoking (those with *p* value  ≤ 9.86 × 10^–06^, distinctly lower than the following sites) were enriched for traits that include Immunoglobulin E (IgE) level, muscle hypertrophy, maternal smoking and birthweight (Fig. 5A). dmCpGs (FDR < 0.05) associated with father’s pubertal smoking were enriched for traits such as autoimmune diseases, atopy, smoking and puberty (Fig. 5B). Enriched traits related to the dmCpGs detected in the EWAS of maternal and personal smoking exposure are provided in Additional file 1: Fig. 1A and 1B for comparison.

### Role of dmCpGs for father’s pubertal smoking (smoking initiation < 15 years)

Given the stronger effects of father’s pubertal smoking than any father’s smoking on offspring DNA methylation than we further explored the biological relevance of these findings.

### Transcription factor enrichment

We interrogated eFORGE TF for transcription factor enrichment in CD4^+^ cells to determine the regulatory role of our 19 significant dmCpGs (FDR < 0.05) related to father’s pubertal smoking. We found significant enrichment of 27 transcription factor binding sites that overlapped with 7 of the dmCpGs (q-value < 0.05) identified in our EWAS study (Additional file 1: Table E9).

### EWAS atlas lookup

**Fig. 6:**
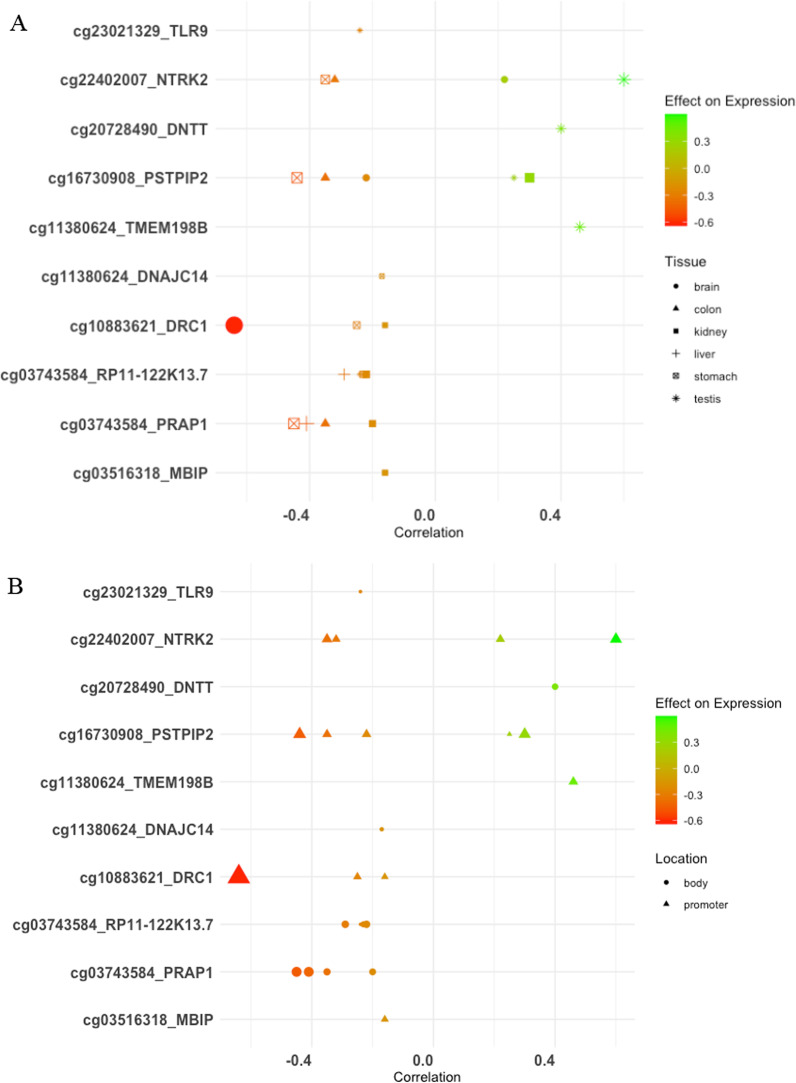
Methylation effects on gene expression regulation across different tissue types for the CpG sites differently methylated according to father’s pubertal smoking starting before age 15 years (FDR < 0.05). [Accessed on 20 June 2021]. Size of point represents − log10 *p* value, colour scale shows CpG site correlation with expression; red to green represents increasing expression. In **A** shape shows the tissue type; in **B** shape shows genomic feature location

Of the 19 dmCpGs associated with father’s pubertal smoking identified in our analysis, 11 were present in the EWAS atlas and correlated with gene expression in a variety of tissues in the EWAS atlas (Fig. 6A) and overlapped with promoters (Fig. 6B) (FDR < 0.05). These were significantly associated with 9 other traits, including atopy and fractional exhaled nitric oxide (cg23021329), smoking (cg20728490; cg16730908), BMI (cg03516318), acute lymphoblastic leukaemia (cg2240207), cancer (cg11380624) and Crohn’s disease (cg10981514) (Additional file 1: Table E10).

### Differentially methylated region (DMR) analysis

No DMRs were significantly associated with father’s any preconception smoking using either DMRcate or dmrff. There were suggestive hits for father’s pubertal smoking, such as DNTT at FDR = 0.084. All DMRs are listed in Additional file 1: Table E11.

### Pathway enrichment

**Fig. 7:**
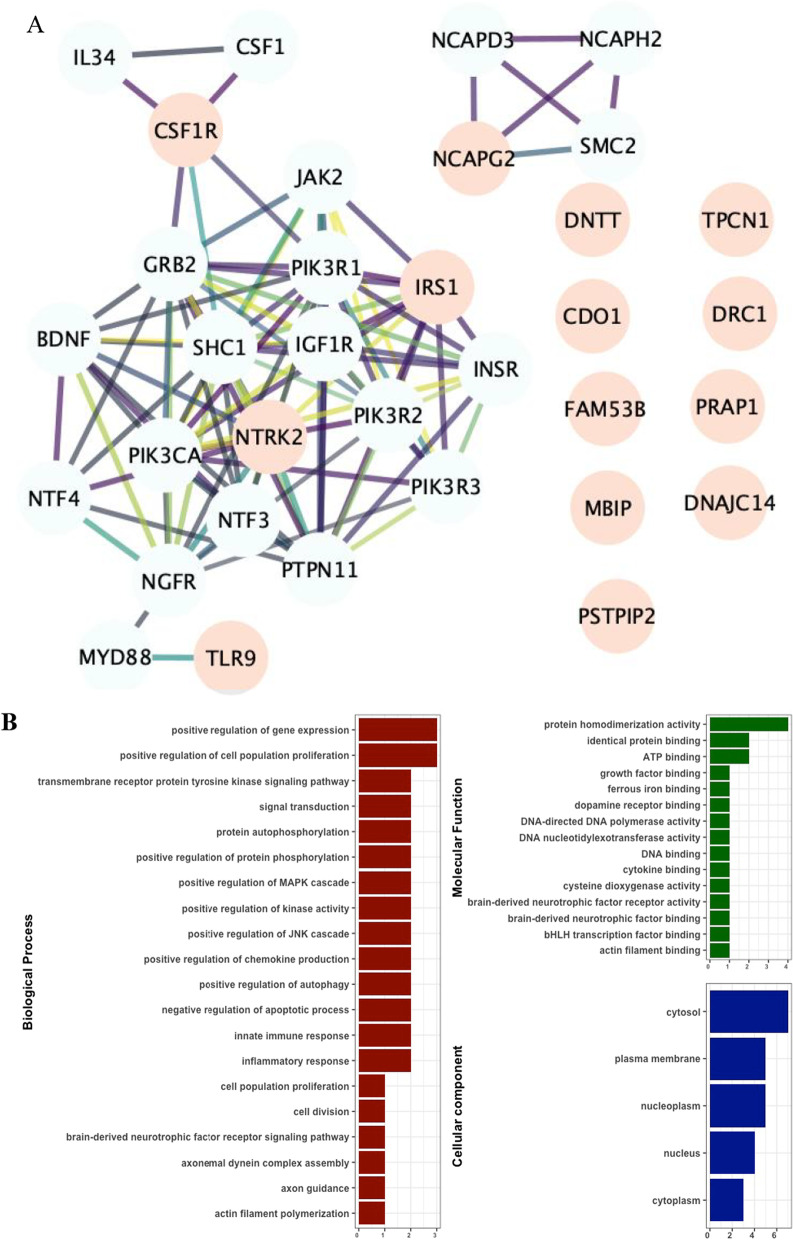
Interactome of dmCpGs associated with father’s pubertal smoking. **A** String network at confidence score 0.7 and 20 top interactors (pale red nodes show dmCpGs, light green nodes show top interactors). The interactions show experimental evidence from score 0.0 (weak) to 1.0 (strong) using the colour gradient yellow (0.06) to deep purple (1.0). **B** Functional enrichment from UniprotKb for the top 15 biological processes, 10 molecular functions and 5 cellular components

To gain further insight into the functional roles of the dmCpGs, we used 14 genes that were mapped to dmCpGs associated with father’s pubertal smoking to generate a protein–protein interaction network from the String database. The top 20 protein interactors were included with high confidence score cutoff 0.7 from protein–protein interaction data sources including experimentally validated protein physical complexes, curated databases and co-expressions. The network indicated that immune response-related genes *TLR9*, *CSF1R*, *NTRK2*, *PTPN11* and *IL34* were well connected (Fig. 7A) (*p* value  < 1.0 × 10^−16^). The molecular function enrichment analysis showed enrichment for gene expression, inflammatory response, innate immunity and cytokine binding (Fig. 7B). We also assessed enrichment of GO terms using gometh. The most significantly enriched biological process terms (FDR < 0.05) include: Inactivation of MAPK activity involved in osmosensory signalling pathway (GO:0000173), negative regulation of interleukin-6 production (GO:0032715), regulation of mast cell chemotaxis (GO:0060753), regulation of neutrophil migration (GO:1902622) and insulin processing (GO:0030070) (Additional file 1: Table E12).

### Replication of DNA methylation signatures associated with father’s preconception smoking

The replication cohort in ALSPAC included 542 participants (female = 280, male = 262), of whom 86 had a father who started to smoke before the age of 15 and 456 had never smoking fathers. There was no overlap of dmCpG sites significantly associated with father’s smoking before age 15 between the two cohorts (FDR < 0.05). However, of the 19 significant dmCpGs identified as related to father’s pubertal smoking in RHINESSA, 11 showed nominal replication in ALSPAC (p < 0.05) with similar direction. The correlation of effects between studies is R = 0.49. The binomial sign test showed the association to be significant at p < 0.05. Expanding the comparison to the top 100 dmCpGs in RHINESSA, the correlation of effects between studies, R = 0.54, *p* value = 3.04 × 10^–05^.

### Sensitivity analyses

There was no association between fathers’ educational level and top dmCpGs identified in relation to fathers’ preconception smoking. There was only weak correlation between father’s smoking dmCpGs and offspring age (maximum R =|0.2|, with 9 CpGs R = 0). In contrast, as expected, the age-related CpG markers showed a strong correlation with age (R > =|0.6|) (Additional file 1: Fig. 2A, B). The study power is shown in Additional file 1: Table E13.

## Discussion

To our knowledge, this is the first human study to investigate the potential epigenetic mechanisms behind the impact of father’s pubertal smoking on offspring. In this epigenome-wide association study, using data from two generations of study participants, we found differentially methylated CpG sites in offspring associated with father’s preconception smoking. Signatures related to father pubertal smoking (smoking initiation before age 15) were much more pronounced than smoking starting at any time preconception. Sixteen of the 19 identified dmCpGs have not previously been reported to be associated with personal or maternal smoking. We suggest these new smoking-associated methylation biomarkers may be specific to smoking exposure of future fathers in early puberty. Several top dmCpGs were enriched for promoter regions and overlapped with significant transcription factor sites that correlated with gene expression in a variety of tissues. Besides unique sites identified for father’s preconception smoking onset, our study confirms previously reported DNA methylation sites associated with personal and mother smoking, demonstrating the validity of our cohort and analytical methods. The genes to which dmCpGs map are related to regulation of innate immunity and inflammatory responses.

For father’s any preconception smoking, we found two novel CpG sites that were not previously linked with any previously investigated smoking phenotype. PRR5 (mapped with cg008870527) is a component of the (mTOR) complex 2 which is upstream of major pathways known to have a crucial role in metabolic regulation and is suggested to play a role in obesity and the pathogenesis of insulin resistance [45]. CENPP (mapped with cg08541349), has been associated with lung function, leucocyte count, BMI and type II hypersensitivity reaction in GWAS studies [38]. Sex-stratified EWAS analyses detected four male-specific dmCpGs that mapped to genes associated with vital capacity (*KCNJ1*, *MYADML2*), IgE levels (relevant to asthma pathogenesis) [46], as well as to genes linked with low-density lipoprotein measurement/ total cholesterol (*TRIM2*) and BMI-related phenotypes (*GRAMD4*, *MYADML2*, *KCNJ1*). In female offspring, we found one dmCpG annotated to *LEPROTL1*, a gene with roles in lung function (FEV1/FVC ratio), growth hormone regulation and glucose homeostasis [47]. Yet, whether male and female offspring in fact display methylation differences at various sites and genes needs further investigation and is yet to be confirmed.

For father’s pubertal smoking, two of our 19 significant CpG sites, have previously been associated with personal smoking (cg20728490 in *DNTT* and cg16730908 in *PSTPIP2*), and they map to genes with important roles in innate immune responses to infections [48, 49]. Upregulation of *PSTPIP2* has also been linked to neutrophilic airway inflammation and non-allergic asthma. When exploring the biological impact of other genes mapped to the dmCpGs uniquely associated with father’s pubertal smoking, several were related to genes associated with innate immunity, allergic diseases and asthma development, such as *TLR9*, *CSF1R*, *DNAJ14*, *NTRK2* and *TPCN1* [48–53]. We also identified CpGs and genes with links to obesity (*NTRK2*, *PSTPIP2*, *MBIP*) [38, 54, 55] and glucose and fat metabolism (*IRS1*). The differentially methylated CpGs were mainly located in open-sea genomic features and enriched for promoter regions, CpG island and gene bodies. These findings suggest that the identified DNA methylation differences, even though of relatively small magnitude, have functional implications in terms of a regulatory role in specific gene expression. Pathway analysis and molecular function enrichment further found interconnection of immune response-related genes and enrichment for inflammatory response, innate immunity and cytokine binding. When seeking replication of results in an independent sample in the ALSPAC, although no dmpCpGs overlapped in the two population cohorts, results showed that effect estimates associated with fathers’ preconception smoking were moderately correlated and with concordant directional effects.

Several mechanistic reports have demonstrated that the toxicogenic components in cigarette smoke impact on epigenetic germline inheritance and affect the offspring’s metabolic health [56]. However, given this is the first study that investigated DNA methylation signatures in young and adult offspring in relation to a timing-specific exposure on father’s smoking, there is limited published literature that is directly comparable to our findings. In a pilot study, we previously observed differentially methylated regions associated with father’s ever smoking, among which annotated genes were related to innate and adaptive immunity and fatty acid synthesis [16]. Preconception paternal smoking has been shown to alter sperm DNA methylation [57] and independently increase asthma risk and reduce lung function in the offspring [9], especially if the smoking started before age 15 [7, 9]. The observed association between the dmCpG sites related to father’s early onset smoking, and offspring asthma, wheezing and weight, suggests that epigenetic changes may lie on the casual pathway between paternal smoke exposures and offspring health outcomes.

Strikingly, the dmCpG sites we identified as related to fathers’ preconception smoking (any preconception smoking as well as pubertal smoking) were quite unique and not the same as those previously reported or found in our data to be associated with mothers’ or personal smoking. As several of the identified CpG sites also are present on the lower coverage 450K array (485512 CpG sites), as shown in Additional file 1: Table E8, the novelty of the identified paternal smoking-associated sites cannot be accounted for by the utilisation of the more comprehensive EPIC methylation array (866838 CpG sites). Reassuringly, our EWAS of mother’s smoking and personal smoking, identified several of the dmCpG sites previously associated with these exposures in other cohorts.

Available data for appropriate replication of our results is a major challenge. We found moderate correlation between RHINESSA and ALSPAC EWAS for paternal smoking before 15 years. Although the replication analysis found effect estimates to have concordant directions in several of the dmCpGs, we did not identify overlapping significant dmCpGs associated with fathers’ preconception smoking in the replication cohort. The low sample size in both cohorts for paternal smoking before 15 might contribute to the lack of shared genome-wide significance. Even within the same population, using different platforms can cause difficulties with replication [58]. The similarity in the direction of association suggests a potential biological effect of early prepuberty father’s smoking, but further research is warranted in order to verify our novel results.

Although we accounted for personal and mother’s smoking exposure in the analysis, we cannot disregard potential residual confounding related to maternal and personal smoking. Further, our analyses cannot fully disentangle effects of father’s early onset smoking from effects of subsequent accumulating second hand smoke exposure. However, epidemiological analyses of various measures of father’s smoking as related to offspring phenotype in over 20,000 father-offspring pairs found that effects of any other aspect of father’s smoking was negligible as compared to that of starting smoking early [7]. We did not control for genetic variation at single nucleotide polymorphisms and cannot rule out that the differentially methylated CpG sites are affected by, or interact with, genetic variants. Our study may be additionally constrained by factors attributable to that of shared familial environments. Although we found no evidence that our top differentially methylated signals were related to fathers’ educational level in a sensitivity analysis, there may be other unmeasured aspects related to social class, which may have influenced our findings. However, a recent analysis of our study cohorts using highly advanced statistical probabilistic simulations demonstrated that unmeasured confounding had a limited impact on the effects of father’s preconception smoking on offspring asthma [8]. This suggests that the identified dmCpGs associated with father’s preconception smoking, most likely are not driven by unmeasured confounding—by genetic factors or by lifestyle-related or environmental factors.

Self-reporting of smoking is another limitation of our study. However, based on validation studies there is an overall consensus that self-report provides a valid and reliable tool for assessing smoking behaviour in cohort studies. Furthermore, it is likely that error in father’s reporting of smoking habits is independent of DNA methylation measured in the offspring, and that misclassification thus will have attenuated the observed results and that the underlying true results might be stronger [59, 60].

We suggest that the observed association between father’s preconception smoking and offspring DNA methylation marks could be caused by transmission through germline imprint of male sperm. Supported by previous mechanistic and epidemiological findings we also speculate that our novel results reflect that early adolescence may constitute a period of particular vulnerability for smoking exposure to modify the offspring’s epigenome. A recent study demonstrated that preconception paternal cigarette exposure in mice from the onset of puberty until 2 days prior to mating modified the expression of miRNAs in spermatozoa and influenced the body weight of F1 progeny in early life [61]. As prepubertal years as well as the onset of puberty represents periods of epigenetic reprogramming events [62], we suggest early adolescence may be a critical time for tobacco-related exposures to interfere with germline epigenetic patterns. This is, however, challenging to study in humans and multiple scientific approaches are needed to elucidate the molecular mechanisms underlying the current findings as well as previous epidemiological results.

## Conclusion

We have identified dmCpG sites in offspring associated with father’s onset of smoking before conception, with most pronounced effects when the father started to smoke already in early puberty (before the age of 15). The pattern differed from those of maternal smoking in pregnancy and of personal smoking, and we suggest these may be unique methylation signatures specific to father’s early adolescent smoking. The genes to which the identified dmCpGs map, are related to asthma, IgE and regulation of innate immunity and inflammatory responses. Our study provide evidence for an epigenetic mechanism underlying the epidemiological findings of high risk of asthma, obesity and low lung function following father’s early adolescent smoking. The functional links of hypermethylated genes suggest that particularly father’s pubertal smoking can have cross-generational effects impacting on the long-term health in offspring. Smoking interventions in early adolescence may have implications for better public health, and potential benefits, not only for the exposed, but also for future offspring.

### Supplementary Information

**Additional file 1.** Supplementary methods and data.

## Data Availability

Summary statistics for the epigenome-wide association analyses reported in the manuscript are available from https://doi.org/10.5258/SOTON/D2566. The underlying data are available on reasonable request. Requests for access to data can be made to the RHINESSA steering committee by PI Professor CS (cecilie.svanes@uib.no) or vice PI VS (vs@ph.au.dk). Reuse of the data must be done in collaboration with the RHINESSA study team. Further information including issues on data security and sharing of data can be found at www.rhinessa.net.
